# Editorial: Epidemiology, diagnosis, prognosis and treatment of rare immune-mediated diseases of the central nervous system

**DOI:** 10.3389/fneur.2023.1342817

**Published:** 2023-12-13

**Authors:** Barbara M. P. Willekens, Ilka Kleffner, Beatrijs Wokke

**Affiliations:** ^1^Department of Neurology, Antwerp University Hospital, Antwerp, Belgium; ^2^Department of Translational Neurosciences, Faculty of Medicine and Health Sciences, University of Antwerp, Antwerp, Belgium; ^3^Laboratory of Experimental Hematology, Faculty of Medicine and Health Sciences, Vaccine and Infectious Disease Institute, University of Antwerp, Antwerp, Belgium; ^4^Department of Neurology, University Hospital Knappschaftskrankenhaus, Ruhr University Bochum, Bochum, Germany; ^5^Department of Neurology, Erasmus Medical Center, Rotterdam, Netherlands

**Keywords:** neuromyelitis optica spectrum disorder (NMOSD), MOGAD, rare disease, neurosarcoidosis, GFAP, systemic disorders, autoimmune encephalitis (AIE)

## Introduction

Rare immune-mediated disorders of the central nervous system (CNS) continue to pose challenges in diagnosis, prognosis and treatment, stressing the importance of sharing knowledge in the research community. In this Research Topic, we aimed to bring together clinical and case studies, epidemiological studies and reviews covering a variety of rare CNS immune-mediated disorders, including autoimmune encephalitis (AIE), neuromyelitis optica spectrum disorders (NMOSD), MOG antibody associated disease (MOGAD), Glial Fibrillary Astrocytic Protein (GFAP) autoimmune astrocytopathy and neurological involvement in systemic disorders such as lupus, rheumatoid arthritis, sarcoidosis and Sjögren's disease.

## Autoimmune encephalitis: clinical findings and prognostication

In this Research Topic, several case reports (Ding C. et al., Khojah et al., Li et al.) address novel findings in autoimmune encephalitis (AIE), including a familiar case of LGI-1 AIE presented by Ding C. et al., suggesting a genetic background and advocating for Genome Wide Association Studies to discover the presence of risk alleles. Li et al. report a case of a patient with anti-GAD65 AIE following HPV vaccination, considering this temporal relationship as a trigger for development of AIE. Finally Khojah et al. have performed a systematic review, including a case vignette on mGluR-1 AIE, stressing the importance of the association of this antibody with cerebellar encephalitis and normal brain imaging in half of patients.

Two manuscripts concerned prognostication. Wu et al. analyzed four models to predict intensive care unit (ICU) admission of patients with AIE in a cohort of 234 patients of whom 40 were admitted to the ICU. The clinical assessment scale in autoimmune encephalitis (CASE) scale plus model, including prodromal symptoms, elevated fasting blood glucose and elevated cerebrospinal fluid (CSF) white blood cell (WBC) count, was selected as the best predictive model. The findings of this model should be externally validated. Ding J. et al. studied 34 patients with anti-gamma-aminobutyric-acid type B receptor (anti-GABA_B_R) encephalitis and found that pulmonary infection and baseline mRS scores were independent risk factors for poor prognosis after a firstline immunotherapy. Finally, Bai et al. report the clinical spectrum, response to immunotherapy and outcomes of patients (*n* = 55) with GAD65 antibodies. The most frequent clinical syndromes were limbic encephalitis (*n* = 34, 61.82%), stiff-person syndrome (SPS; *n* = 18, 32.73%), cerebellar ataxia (*n* = 11, 20%) or overlap syndromes. Almost 60% of patients had other autoimmune conditions, including Hashimoto thyroiditis, type 1 diabetes mellitus and vitiligo. A minority (*n* = 2, 3.64%) of patients had underlying tumors, including thymoma and small cell lung carcinoma. Most patients had short-term favorable outcomes with Modified Ranking Scale ≤ 2 (87%). Longterm outcomes showed more variation and were dependent on the clinical phenotype.

## Clinical presentation, prognostication and management of NMOSD, MOGAD and GFAP autoimmune astrocytopathy

GFAP antibodies were first described in 2016 (1) as a biomarker of relapsing meningoencephalomyelitis. Over the years, the clinical spectrum has extended. Zhu et al. report on 59 adults and children with GFAP antibodies in serum or CSF of whom 55 were positive only in the CSF. Interestingly, in almost a quarter of them multiple autoantibodies were detected, most frequently AQP4 antibodies. The most common phenotype in children was encephalomyelitis (9/18, 50%) and in adults encephalitis (15/41, 36.6%). More than 80% had a monophasic course over a median followup time of 9 months. Zhang, Xie et al. performed a similar retrospective analysis of 33 patients, with a slightly longer median followup time of 12 months, reporting relapses while steroids were tapered in four patients. Almost 80% had good outcomes in the short-term. A study by Sun et al. compared clinical and imaging features of GFAP and MOG antibody associated myelitis in 14 and 24 patients respectively, in order to differentiate these disorders. Higher protein CFS levels were found in GFAP vs. MOG antibody positive patients, which may help clinicians differentiate these diseases.

While many patients with NMOSD have a good response to rituximab, some may be none-responders. Zhang, Jiao et al. report a difficult to treat NMOSD case and present a successful treatment approach with ofatumumab and IVIg.

The intriguing observation and role of enlarged perivascular spaces in NMOSD is discussed by Yao et al. while a temporal association of NMOSD with SARS-CoV-2 vaccination is discussed in a systematic review by Harel et al.. The often difficult patient journey from diagnosis to chronic disease is well-described by Delgado-Garcia et al..

## Neurological involvement in systemic disorders

Finally, some interesting cohorts are presented, discussing the clinical presentation, diagnostic approach and management of neurosarcoidosis (Sambon et al.), rheumatoid meningitis (Fan et al.), and Sjögren's syndrome (Hoshina et al.). A cohort of patients with MRI negative myelitis, show that this can be a presenting feature of lupus (Das et al.).

## Concluding remarks

Overall, this Research Topic includes recent and emerging insights on clinical aspects of rare CNS immune-mediated disorders.

## Author contributions

BMPW: Conceptualization, Writing—original draft, Writing—review & editing. IK: Writing—review & editing. BW: Writing—review & editing.

## References

[1] Fang B McKeon A Hinson SR Kryzer TJ Pittock SJ Aksamit AJ. Autoimmune glial fibrillary acidic protein astrocytopathy: a novel meningoencephalomyelitis. JAMA Neurol. (2016) 73:1297–307. 10.1001/jamaneurol.2016.2549 27618707

